# Vestibular Schwannoma Presenting as Oral Dysgeusia: An Easily Missed Diagnosis

**DOI:** 10.1155/2016/7081919

**Published:** 2016-02-18

**Authors:** Emma Brown, Konrad Staines

**Affiliations:** ^1^Oral Medicine Department, Bristol Dental Hospital, Lower Maudlin Street, Bristol BS1 2LY, UK; ^2^Oral Medicine Department, University of Bristol Dental School, Lower Maudlin Street, Bristol BS1 2LY, UK

## Abstract

We present a case of a fifty-year-old male patient who was referred to the Oral Medicine Department with a complaint of a salty taste. History taking subsequently revealed that the patient was also experiencing intermittent numbness of his left lower lip, tinnitus, and a feeling of fullness in the left ear. Magnetic resonance imaging was performed which revealed a large vestibular schwannoma affecting the left vestibulocochlear nerve, which was treated surgically. This case shows the importance of taking a detailed history in a patient presenting with an initial complaint of oral dysgeusia. It also highlights the possibility of significant underlying pathology, presenting with initial low level, nonspecific complaints such as an altered taste, and the rationale for imaging patients who report unilateral facial hypoesthesia.

## 1. Introduction

Oral dysgeusia is defined as a gustatory disturbance relating to a distorted taste perception, or to a persistent taste sensation in the absence of stimulation [1], and can be very distressing for the patient. Patients with taste disturbances are commonly referred to and investigated within Oral Medicine Departments in the UK. Identifiable causes of dysgeusia can include local factors, drugs, systemic disease, and peripheral or central neurological conditions; see Table 1 [1–5].

Local factors influencing taste include infections of the oral cavity, such as candidiasis or dental abscess, trauma, or hyposalivation [1–3]. Over 250 drugs have been associated with oral dysgeusia, of which the most common are outlined in Table 1 [3–5]. Dysgeusia can also be a manifestation of a systemic disease such as an underlying haematinic deficiency, diabetes mellitus, Crohn's disease, or Sjögren's syndrome [1, 3]. Rarely, damage to the peripheral or central nervous system can be the cause. Tumours affecting the cerebellopontine angle, such as a schwannoma or meningioma, can cause taste disturbances. Central neurological causes such as ischaemia, haemorrhage, or demyelination affecting the gustatory pathway in the brain, or temporal lobe epilepsy, also require exclusion [1].

In a significant number of cases, the taste disturbance cannot be attributed to an identifiable cause and therefore a diagnosis of idiopathic oral dysgeusia is made. Although altered taste is a rare symptom of vestibular schwannoma, this case illustrates the need for a high index of clinical suspicion when assessing patients with seemingly low level symptoms, such as oral dysgeusia.

## 2. Case Presentation

### 2.1. History

A fifty-year-old male patient was referred to the Oral Medicine Department by his General Dental Practitioner with a primary complaint of an altered taste. The taste disturbance had been affecting the patient for six months and manifested as a constant salty taste. His General Medical Practitioner had empirically prescribed omeprazole to exclude gastric oesophageal reflux disease as a potential cause. However, this was ineffective and therefore the patient had discontinued the treatment.

Additionally, the patient also complained of a cracked tongue surface and intermittent numbness of his lower left lip and chin. He had experienced three episodes of facial hypoesthesia affecting the mandibular division of the left trigeminal nerve, which had all been self-limiting and resolved spontaneously.

Medical history was clear except for tinnitus and a feeling of fullness affecting the left ear for the past three years. This had been diagnosed by the General Medical Practitioner as Eustachian tube dysfunction and treated with fluticasone nasal spray. This had no benefit in relieving the symptoms and was discontinued. The patient therefore took no regular medication.

Socially, the patient was a semiretired office based worker. He was a nonsmoker and had a low alcohol intake.

### 2.2. Examination

Cranial nerve examination at the initial clinic appointment did not reveal any sensory disturbance along the trigeminal nerves or motor deficit of the facial nerves. The eyes were unaffected, with no lacrimation abnormalities identified. Swallow and phonation were not affected. The uvula was not deviated, and the movement of the palate and tongue was normal. Extra oral examination of the head and neck showed normal anatomy, with no facial swelling and no cervical lymphadenopathy. Intraorally the soft tissues of the mouth were healthy with no mucosal lesions detected and no obvious source of infection that could cause the oral dysgeusia. The cracked tongue surface was deemed to be a variation of normal anatomy termed a fissured tongue.

### 2.3. Investigations

An orthopantomogram radiograph was completed, which confirmed the absence of dental or bony pathology. Blood investigations, including a full blood count, blood glucose, serum ferritin, folate, and vitamin B12 levels, all resulted as normal.

**Figure 1:**
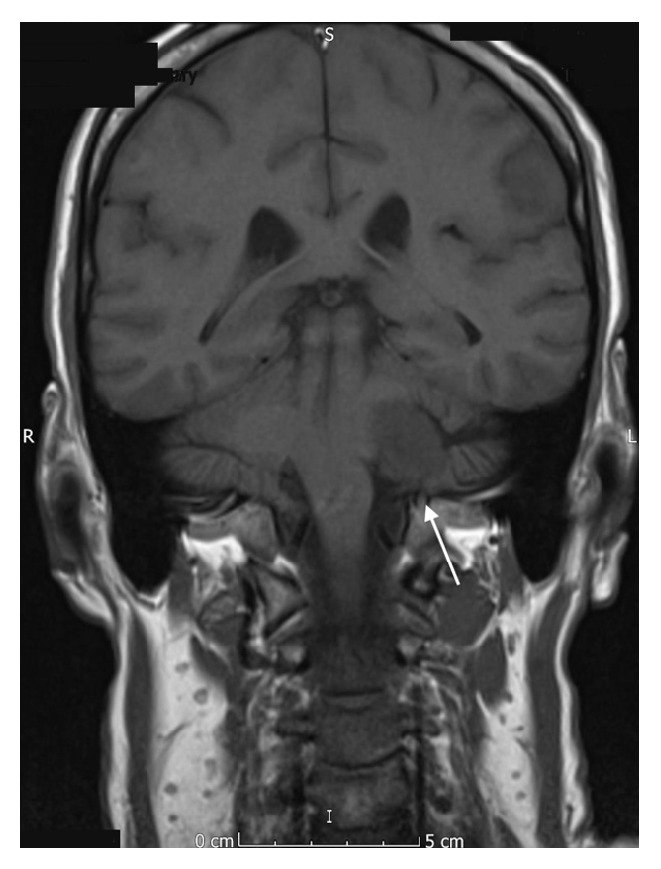
Coronal MR image (T1 weighted) showing the vestibular schwannoma affecting the left vestibulocochlear nerve.

**Figure 2 fig2:**
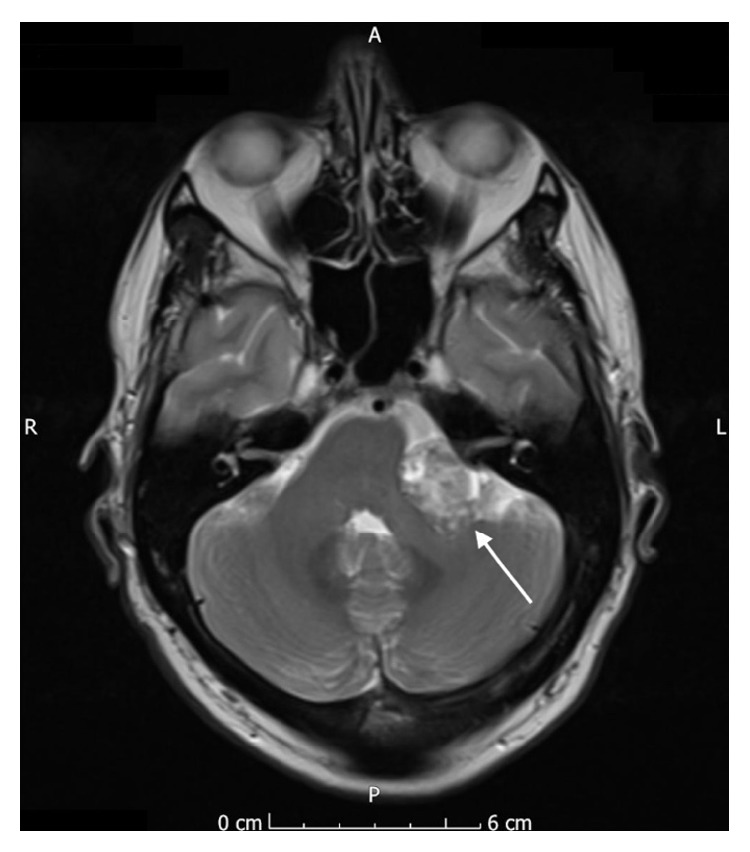
Axial MR image (T2 weighted, fat saturated) showing the vestibular schwannoma affecting the left vestibulocochlear nerve.

**Figure 3 fig3:**
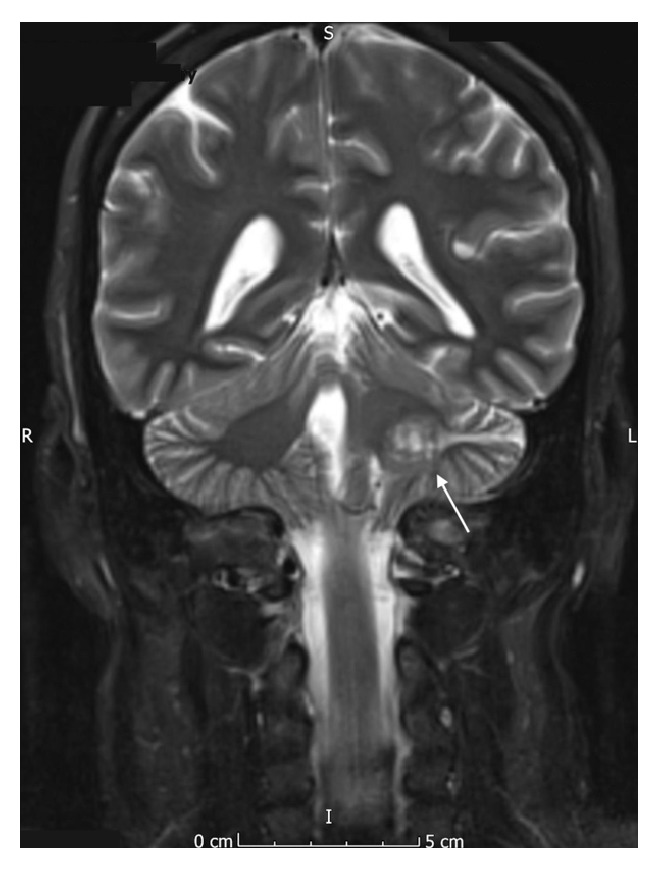
Coronal MR image (T2 weighted, fat saturated) showing the vestibular schwannoma affecting the left vestibulocochlear nerve.

**Figure 4:**
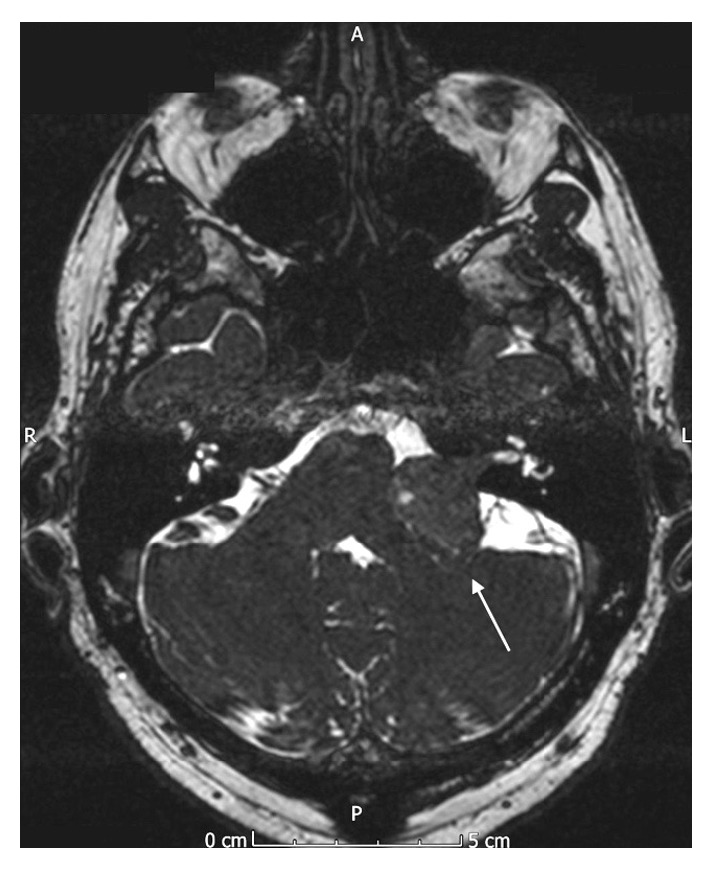
Axial MR image (T2 diffusion-weighted image) showing the vestibular schwannoma affecting the left vestibulocochlear nerve.

Due to the reported facial hypoesthesia, magnetic resonance imaging of the head was performed. This identified a large vestibular schwannoma of the left vestibulocochlear nerve (eighth cranial nerve). This measured 2.7 cm by 2.4 cm by 2.3 cm and was located in the left cerebellopontine angle, involving the left internal auditory meatus. There was associated compression of the brainstem and distortion of the fourth ventricle, without hydrocephalus (see Figures 1 [Fig fig2] [Fig fig3]–4).

### 2.4. Treatment

The patient was referred to the Ear, Nose, and Throat Team for further management. They identified 45 to 50 decibels of sensorineural hearing loss in the left ear on audiogram. There was also some instability on standing, particularly when the patient's eyes were closed. The hypoesthesia of the left trigeminal nerve had worsened, with a constant tingling affecting the maxillary and mandibular divisions and reduced pin prick sensation in these regions.

Treatment options for vestibular schwannoma include monitoring with serial scanning to determine extent of tumour growth, microsurgery to remove or debulk the tumour, or stereotactic radiosurgery [6]. Following discussion at the Skull Base Multidisciplinary Team Meeting, which included a Clinical Oncologist, Neuroradiologist, Ear, Nose, and Throat Surgeon, and Neurosurgeon, a decision was made that microsurgery would be the best treatment option. This was due to the large size of the vestibular schwannoma, the neurological symptoms, and the associated compression of the brain stem. Therefore, the patient underwent surgical excision to remove the tumour and decompress the brainstem. This was undertaken in a joint case with the Ear, Nose, and Throat and Neurosurgery Teams. A translabyrinthine approach was used, which leads to complete hearing loss on the affected side but aims to preserve the facial nerve.

### 2.5. Outcome

Surgical treatment was successful and the histology showed a schwannoma comprising densely cellular Antoni A areas with less densely cellular Antoni B areas. There was no significant mitotic activity or necrosis. Immunohistochemistry showed that it was strongly positive for S100. The conclusion was that it was a benign schwannoma of the left cerebellopontine angle, WHO Grade I. These results were discussed postoperatively at the Skull Base Multidisciplinary Team Meeting and it was agreed that the patient would be followed up by the Neurosurgery Team.

The patient is doing well postoperatively. The aural fullness in the left ear has now completely resolved, and the tinnitus is barely noticeable. The facial hypoesthesia that affected the maxillary and mandibular divisions of the left trigeminal nerve preoperatively has also completely resolved. There was some minor facial weakness, House-Brackmann Grade II, which was present immediately after surgery and has now returned to normal. Furthermore, the oral dysgeusia has improved since the surgery, with an occasional salty taste still noticed. The patient is now awaiting CROS hearing aids to help with his hearing loss on the left side. His balance was severely affected from the surgery but this has now significantly improved.

**Figure 5:**
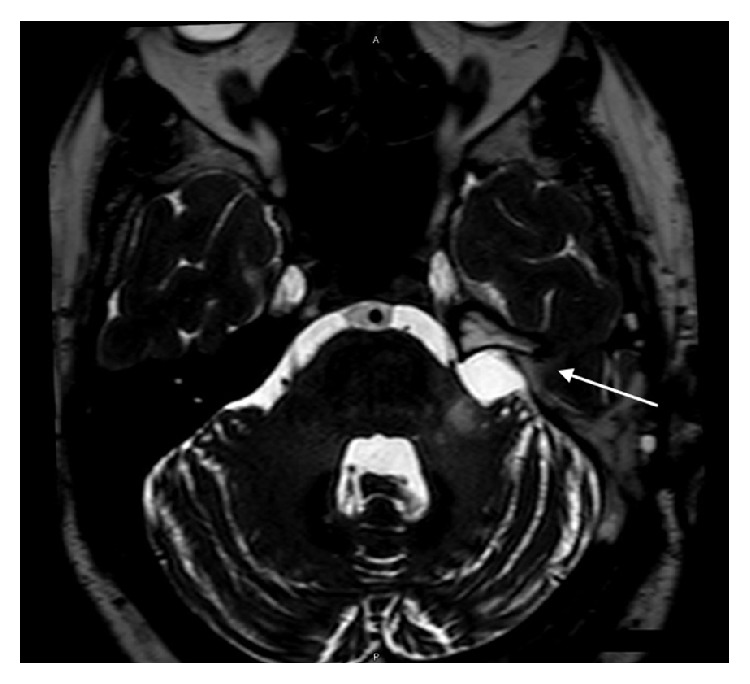
Axial MR image (T2 weighted, 3D DRIVE CLEAR) showing the postoperative changes.

**Figure 6 fig6:**
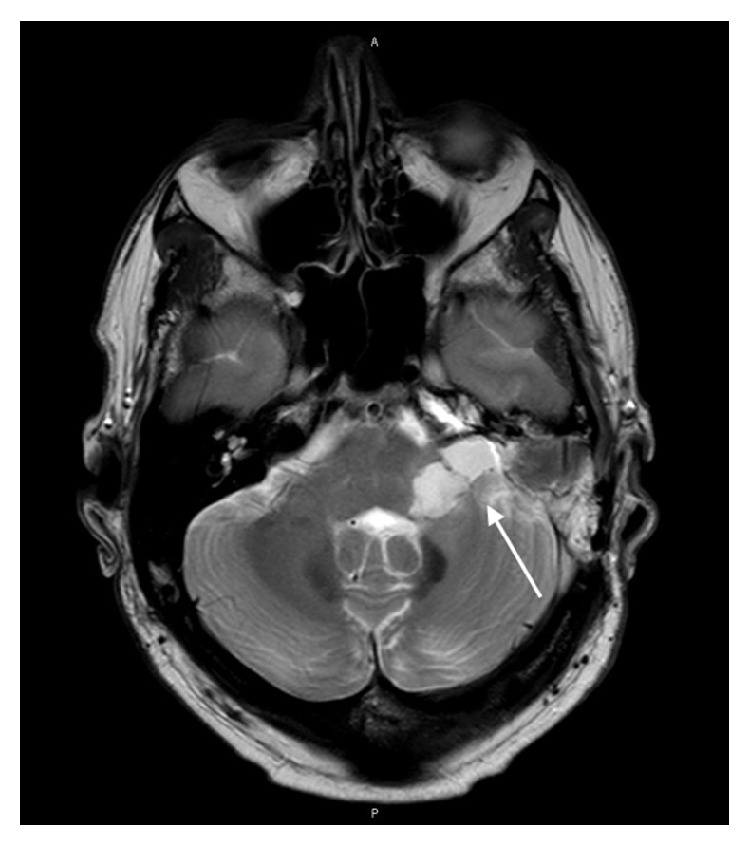
Axial MR image (T2 weighted) showing the postoperative changes.

**Figure 7:**
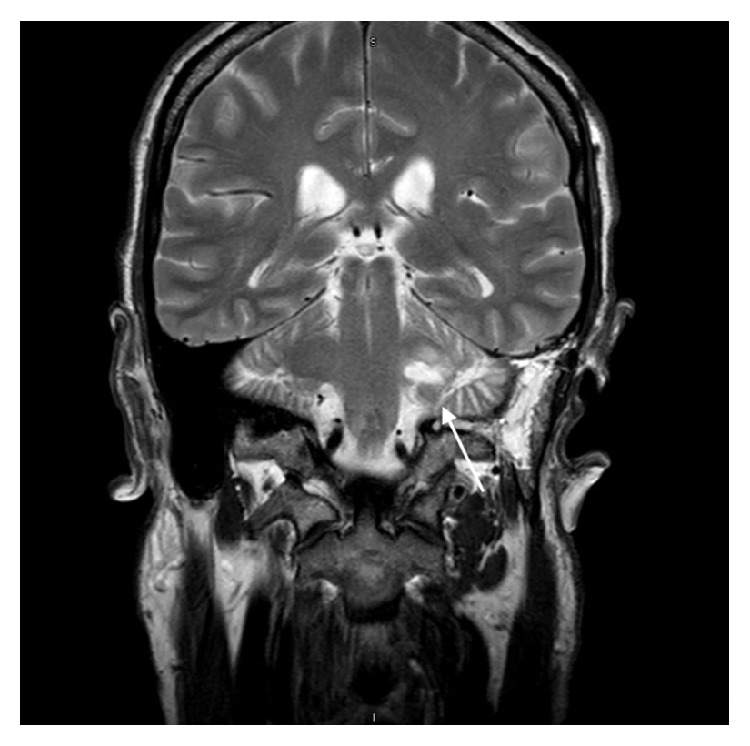
Coronal MR image (T2 weighted) showing the postoperative changes.

Postoperative magnetic resonance imaging was performed at six months postoperatively (see Figures 5 [Fig fig6]–7). The figures show normal postsurgical changes involving the petrous bone and cerebellopontine angle. The left sided vestibular schwannoma has been resected. There is a cavity in the left middle cerebellar peduncle which is likely to represent an area of perioperative ischaemia. There is a thin cuff of residual tissue anterior to the site of the cerebellopontine angle tumour. The remnant of the schwannoma will be monitored by the Neurosurgery Team with a further magnetic resonance imaging in one year.

## 3. Discussion

Vestibular schwannomas, also termed acoustic neuromas, are benign tumours of the vestibulocochlear nerve [7–14]. They arise from the Schwann cells which comprise the myelin sheath surrounding the vestibular branch of the eighth cranial nerve [14] and account for 80–90% of cerebellopontine angle tumours [7, 8, 11, 12]. The aetiology of vestibular schwannoma is unknown. The most common symptoms are unilateral hearing loss, tinnitus, or unsteadiness [7–13]. As the tumour grows it compresses surrounding intracranial structures. Compression of the trigeminal nerve can lead to facial hypoesthesia, which is found in 7–26% of patients with vestibular schwannoma [9, 15], and usually begins in the region of the anterior mandible [9]. In 2.2–17% of patients the facial nerve is affected, due to the close anatomical relationship of the facial nerve to the vestibulocochlear nerve in the region of the cerebellopontine angle [9, 11, 15]. Pressure on the motor fibres can lead to facial weakness; and involvement of the sensory branch, the nervus intermedius which receives special sensory taste information from the anterior two-thirds of the tongue, via the chorda tympani, can have an impact on taste [15, 16]. There are a number of studies reporting facial numbness as the presenting feature of a vestibular schwannoma [7, 8, 12]. In another case, the patient presented with a burning sensation of the tongue [14], but no other report has described oral dysgeusia as the symptom prompting referral to secondary care.

Facial nerve dysfunction is rarely the presenting symptom of vestibular schwannoma [13]. However, literature shows that, in up to 17% of patients, the facial nerve is affected [15]. When the facial nerve is compressed by the tumour, taste disturbances appear earlier than facial palsy. This is because sensory nerves are more sensitive to compression than motor fibres [16]. A study by Watanabe et al. [17] found that preoperative taste disturbances were found in 28.7% of patients undergoing surgery for a vestibular schwannoma. Another study by Sahu et al. [16] tested 142 patients with vestibular schwannoma and found taste disturbances present in 40.8% of patients preoperatively. Matthies and Samii [15] found that taste disturbances were only reported in 1.8% of 1000 patients diagnosed with vestibular schwannoma, although they did not formally test this and assumed that the actual incidence would be higher. They also established that preoperative taste disturbance was a reliable indicator of facial nerve compromise and impending paresis.

Although oral dysgeusia is rarely the presenting symptom, it is clear that a significant proportion of patients with vestibular schwannoma do experience taste disturbances. It is therefore imperative that for patients presenting with altered taste a detailed history is taken. The history should specifically include questions relating to sensory loss, and the appropriate investigations should be performed to rule out any significant underlying pathology.

## Figures and Tables

**Table 1:** Possible causes of oral dysgeusia [1–5].

Local factors	Dental abscess, candida infection, trauma to the oral cavity, hyposalivation, removable prostheses, and gastric oesophageal reflux disease

Drugs	Antimicrobials
	Antibacterials: cephalosporins, ciprofloxacin, clarithromycin, clindamycin, co-trimoxazole, doxycycline, imipenem with cilastatin, metronidazole, ofloxacin, penicillins, trimethoprim, tetracycline, and vancomycin
	Antivirals: aciclovir, ganciclovir, indinavir, ritonavir, and zidovudine
	Antifungals: fluconazole and griseofulvin
	Central nervous system
	Antiparkinsonians: bromocriptine, levodopa, and pergolide
	Migraine medications: naratriptan and sumatriptan
	Muscle relaxants: baclofen and methocarbamol
	Tricyclic antidepressants: amitriptyline, clomipramine, desipramine, doxepin, imipramine, nortriptyline, and trimipramine
	Selective serotonin reuptake inhibitors: citalopram, fluoxetine, fluvoxamine, and sertraline
	Antipsychotics: clozapine, olanzapine, and risperidone
	Benzodiazepines: alprazolam, clonazepam, flurazepam, and midazolam
	Mood stabilisers: lithium
	Cardiovascular
	Angiotensin-converting enzyme inhibitors: captopril, enalapril, lisinopril, and perindopril
	Beta-adrenoreceptor blockers: labetalol, metoprolol, propranolol, and timolol
	Calcium-channel blockers: amlodipine, diltiazem, and nifedipine
	Potassium-sparing diuretics: amiloride and triamterene
	Statins: atorvastatin, fluvastatin, and simvastatin
	Antiarrhythmics: amiodarone, flecainide, and procainamide
	Endocrine
	Diabetic medications: glibenclamide, metformin, and tolbutamide
	Other
	Antineoplastics: busulfan, dacarbazine, vinblastine, and vincristine
	Antimalarials: hydroxychloroquine, pyrimethamine, and quinine
	Bisphosphonates: alendronic acid, etidronic acid, and pamidronic acid
	Bronchodilators: salbutamol and terbutaline
	Nonsteroidal anti-inflammatories: celecoxib, diclofenac, ketoprofen, and ketorolac

Systemic disease	Diabetes mellitus, haematinic deficiency (ferritin, folate, or vitamin B12), Sjögren's syndrome, Crohn's disease, and chronic kidney disease

Peripheral nervous system	Syndromes affecting the facial, glossopharyngeal, or vagal nerves (e.g., Bell's palsy) and tumours of the skull base or cerebellopontine angle (meningioma, schwannoma, epidermoid tumour, and brain metastasis)

Central nervous system	Ischaemia, haemorrhage, or demyelination affecting the brainstem, thalamus or cortical components of the gustatory pathway, and temporal lobe epilepsy
